# *Notes from the Field: *Antihistamine Positivity and Involvement in Drug Overdose Deaths — 44 Jurisdictions, United States, 2019–2020

**DOI:** 10.15585/mmwr.mm7141a4

**Published:** 2022-10-14

**Authors:** Amanda T. Dinwiddie, Lauren J. Tanz, Jessica Bitting

**Affiliations:** ^1^Division of Overdose Prevention, National Center for Injury Prevention and Control, CDC; ^2^National Network of Public Health Institutes, Washington, D.C.

Antihistamines are frequently used to treat allergy symptoms (*1*). Misuse of antihistamines has been documented primarily in adolescents and young adults (*2*); however, antihistamine involvement in overdose deaths has not been widely studied. Among the various antihistamine subtypes, the first-generation H1 subtype can cause anticholinergic effects, including strong sedation (*3*) that might be exacerbated when co-used with other sedative drugs (e.g., opioids).* Diphenhydramine, a common over-the-counter first-generation H1 antihistamine, has been combined with opioids as an adulterant^†^ in illicit drug supply (*4*) and can be used to reduce opioid-related side effects (e.g., itchy skin because of histamine release from opioid use).

To describe unintentional and undetermined intent overdose deaths with antihistamine positivity, involvement, or both, CDC analyzed available 2019–2020 data from the State Unintentional Drug Overdose Reporting System (SUDORS) in 43 states and the District of Columbia.^§^^,^^¶^ A death was defined as antihistamine-positive if any antihistamine was detected on postmortem toxicology or was listed as a cause of death on the death certificate.** A death was defined as antihistamine-involved if the drug class was listed as a cause of death on the death certificate (i.e., antihistamine-involved is a subset of antihistamine-positive). Descriptive data for deaths are presented by sex, age, race and ethnicity, U.S. Census Bureau region,^††^ and other drugs involved. Analyses were restricted to decedents for whom postmortem toxicology results were available.^§§^

Among 92,033 overdose deaths during 2019–2020, 13,574 (14.7%) were antihistamine-positive and 3,345 (3.6%) were antihistamine-involved; fewer than 0.1% (90) involved antihistamines alone (Table). Nearly all antihistamine-positive and -involved deaths (13,475, 99.6%; 3,339, 99.8%, respectively) included first-generation H1 antihistamines, primarily diphenhydramine (9,645, 71.1%; 2,226, 66.5%, respectively). The proportions of antihistamine- and diphenhydramine-involved overdose deaths were highest for females (52.0%; 52.8%), persons aged 35–44 years (26.1%; 26.5%), and White, non-Hispanic persons (78.1%; 78.7%); demographic patterns of antihistamine- and diphenhydramine-positive deaths were similar, except that deaths were more frequent among males (57.8%; 59.6%) and in the Midwest region (43.6%; 51.0%). Most antihistamine- and diphenhydramine-involved overdose deaths co-involved opioids (82.8% and 82.7%, respectively), primarily illicitly manufactured fentanyls (IMFs)^¶¶^ (*5*).

**TABLE:** Characteristics of overdose decedents with antihistamine-positive* or -involved results^†^ — State Unintentional Drug Overdose Reporting System, 44 Jurisdictions,^§^ United States, 2019–2020

Characteristic	Classification of deaths, no. (%)			
	Antihistamine-positive (n = 13,574)	Antihistamine-involved (n = 3,345)	Diphenhydramine-positive (n = 9,645)	Diphenhydramine-involved (n = 2,226)
**Sex**				
Male	7,842 (57.8)	1,605 (48.0)	5,745 (59.6)	1,050 (47.2)
Female	5,732 (42.2)	1,740 (52.0)	3,900 (40.4)	1,176 (52.8)
**Age group, yrs** ^¶^				
<18	46 (0.3)	21 (0.6)	26 (0.3)	13 (0.6)
18–24	736 (5.4)	179 (5.4)	498 (5.2)	110 (4.9)
25–34	3,056 (22.5)	685 (20.5)	2,155 (22.3)	422 (19.0)
35–44	3,538 (26.1)	874 (26.1)	2,502 (25.9)	591 (26.5)
45–54	3,151 (23.2)	781 (23.3)	2,219 (23.0)	539 (24.2)
55–64	2,450 (18.1)	637 (19.0)	1,776 (18.4)	427 (19.2)
≥65	596 (4.4)	168 (5.0)	468 (4.9)	124 (5.6)
**Race and ethnicity**				
White, non-Hispanic	9,513 (70.6)	2,589 (78.1)	6,613 (69.0)	1,732 (78.7)
Black, non-Hispanic	2,822 (20.9)	400 (12.1)	2,200 (23.0)	274 (12.4)
Other or multi-race, non-Hispanic**	289 (2.1)	81 (2.4)	179 (1.9)	53 (2.4)
Hispanic	859 (6.4)	244 (7.4)	587 (6.1)	143 (6.5)
Unknown or missing	91	31	66	24
**U.S. Census Bureau region** ^††^				
Northeast	2,439 (18.0)	930 (27.8)	1,487 (15.4)	597 (26.8)
Midwest	5,921 (43.6)	894 (26.7)	4,916 (51.0)	662 (29.7)
South	3,952 (29.1)	909 (27.2)	2,555 (26.5)	596 (26.8)
West	1,262 (9.3)	612 (18.3)	687 (7.1)	371 (16.7)
**Co-involved drugs (listed as a cause of death)** ^§§,¶¶^				
Opioids	11,867 (87.4)	2,771 (82.8)	8,570 (88.9)	1,841 (82.7)
IMFs ***	9,307 (68.6)	1,735 (51.9)	7,012 (72.7)	1,190 (53.5)
Non-IMF opioids	2,560 (18.9)	1,036 (31.0)	1,558 (16.2)	651 (29.2)
Heroin^†††^	3,242 (23.9)	587 (17.5)	2,491 (25.8)	395 (17.7)
Prescription opioids^§§§^	3,676 (27.1)	1,410 (42.2)	2,432 (25.2)	937 (42.1)
Cocaine	3,221 (23.7)	555 (16.6)	2,282 (23.7)	378 (17.0)
Methamphetamine	1,815 (13.4)	365 (10.9)	1,198 (12.4)	235 (10.6)
Benzodiazepines	2,703 (19.9)	1,152 (34.4)	1,786 (18.5)	739 (33.2)
Alcohol	2,044 (15.1)	603 (18.0)	1,484 (15.4)	411 (18.5)
Gabapentin	1,189 (8.8)	642 (19.2)	732 (7.6)	385 (17.3)
**No other drug** ^¶¶¶^	27 (0.2)	90 (2.7)	23 (0.2)	71 (3.2)

**Abbreviations:** IMFs = illicitly manufactured fentanyls; SUDORS = State Unintentional Drug Overdose Reporting System.

* Drug entries coded as antihistamines in SUDORS were allergy, allergy relief, antihistamines, Atarax, Benadryl, brompheniramine, carbinoxamine, cetirizine or cetirizine metabolite, chlorcyclizine or chlorcyclizine metabolite, chlorpheniramine or chlorpheniramine metabolite, cyproheptadine or cyproheptadine metabolite, desalkylhydroxyzine, descarboethoxyloratadine, desloratadine, dexbrompheniramine, dexchlorpheniramine, diphenhydramine or diphenhydramine metabolite, doxylamine or doxylamine metabolite, fexofenadine, hydroxyzine or hydroxyzine metabolite, hydroxyzine/cetirizine/meclizine (not distinguished) metabolite, levocetirizine, loratadine, mereprine, norchlorcyclizine, norcyproheptadine, nordiphenhydramine, norhydroxyzine norpromethazine, Nytol, phenergan, pheniramine, phenyltoloxamine, promethazine or promethazine metabolite, pyrilamine, tripelennamine, triprolidine, Unisom, Vistaril, and Zyrtec.

^†^ SUDORS captures data on fatal unintentional and undetermined intent overdoses. For all captured overdose deaths, SUDORS records all drugs detected by postmortem toxicology, even those not ruled by a medical examiner or coroner as causing death. A drug was recorded as positive when it was detected on postmortem toxicology or listed as a cause of death on the death certificate. A drug was recorded as involved when it was listed as a cause of death on the death certificate. The medical examiner or coroner lists drugs on the death certificate based on any of the following: postmortem toxicology detection, evidence of drug use at the scene, or witness reports of drug use.

^§^ Among 48 funded jurisdictions, 43 states and the District of Columbia reported data during January 2019–December 2020. Twenty-six jurisdictions reported deaths that occurred during the entire period: Alaska, Connecticut, Delaware, District of Columbia, Georgia, Illinois, Kentucky, Maine, Massachusetts, Minnesota, Missouri, Nevada, New Hampshire, New Jersey, New Mexico, North Carolina, Ohio, Oklahoma, Pennsylvania, Rhode Island, Tennessee, Utah, Vermont, Virginia, Washington, and West Virginia. Eighteen additional jurisdictions reported deaths during at least one 6-month period during January 2019–December 2020 (i.e., January–June 2019, July–December 2019, January–June 2020, or July–December 2020): Alabama, Arizona, Arkansas, Colorado, Florida, Hawaii, Indiana, Iowa, Kansas, Louisiana, Maryland, Michigan, Mississippi, Montana, Nebraska, Oregon, South Dakota, and Wisconsin. Fifteen jurisdictions reported deaths from counties that accounted for ≥75% of drug overdose deaths in the state in 2017 for at least one 6-month period per SUDORS funding requirements (Alabama, Arkansas, Colorado, Florida, Hawaii, Illinois, Indiana, Louisiana, Michigan, Missouri, Nebraska, Pennsylvania, South Dakota, Washington, and Wisconsin); all other jurisdictions reported deaths from the full jurisdiction. Data were current as of April 25, 2022.

^¶^ Age data were missing for one decedent.

** Includes non-Hispanic American Indian or Alaska Native, non-Hispanic Asian or Pacific Islander, or non-Hispanic multiracial persons.

^††^ https://www2.census.gov/geo/pdfs/maps-data/maps/reference/us_regdiv.pdf

^§§^ Identified as a cause of death by a medical examiner or coroner.

^¶¶^ Multiple drugs could be listed as a cause of death; therefore, drugs are not mutually exclusive.

*** IMFs include illicitly manufactured fentanyl and illicit fentanyl analogs, which were identified using both toxicology and scene evidence because toxicology alone cannot distinguish between pharmaceutical fentanyl and IMFs.

^†††^ Drug entries coded as heroin in SUDORS were heroin and 6-acetylmorphine. In addition, morphine was coded as heroin if detected along with 6-acetylmorphine or if scene, toxicology, or witness evidence indicated presence of known heroin adulterants or impurities (including quinine, procaine, xylazine, noscapine, papaverine, thebaine, or acetylcodeine), injection, illicit drug use, or a history of heroin use.

^§§§^ Drug entries coded as prescription opioids in SUDORS were alfentanil, buprenorphine, butorphanol, codeine, dextrorphan, dihydrocodeine, hydrocodone, hydromorphone, levorphanol, loperamide, meperidine, methadone, morphine, nalbuphine, noscapine, oxycodone, oxymorphone, pentazocine, prescription fentanyl, propoxyphene, sufentanil, tapentadol, and tramadol. Also included as prescription opioids were brand names and metabolites (e.g., nortramadol) of these drugs and combinations of these drugs and nonopioids (e.g., acetaminophen-oxycodone). Morphine was included as prescription only if scene or witness evidence did not indicate likely heroin use and if 6-acetylmorphine was not also detected. Fentanyl was coded as a prescription opioid based on scene, toxicology, or witness evidence.

^¶¶¶^ Includes antihistamine-positive deaths with antihistamines as only drug positive, antihistamine-involved deaths with antihistamines as only drug involved, diphenhydramine-positive deaths with diphenhydramine as only drug positive, and diphenhydramine-involved deaths with diphenhydramine as only drug involved.

Nearly 15% of overdose deaths during 2019–2020 were antihistamine-positive, and 4% were antihistamine-involved; only 90 deaths involved antihistamines as the sole drug. Most antihistamine-positive and antihistamine-involved deaths included diphenhydramine, which is easily accessible over the counter as an allergy medication and sleep aid. Antihistamine-involved deaths commonly co-involved opioids; this might be partly attributable to adulteration of the illicit opioid supply with antihistamines, in particular diphenhydramine, which can be dangerous because of potentially combined sedative effects. Naloxone administration is important for any overdose with suspected opioid involvement. Because antihistamines do not respond to naloxone, co-involved opioid and antihistamine overdoses might require naloxone administration plus other immediate medical response measures to prevent death.

The findings in this report are subject to at least two limitations. First, results included 44 jurisdictions and might not be nationally representative. Second, drug testing methods are not standardized across jurisdictions, which might limit interpretation of results. Antihistamine-positivity could reflect use to treat allergy or other symptoms rather than misuse. It is also possible that some persons did not knowingly consume antihistamines and were exposed to these drugs through adulteration of the illicit drug supply with antihistamines. Despite these limitations, these data highlight the importance of continued surveillance to understand the drugs and drug combinations contributing to overdose deaths and to guide awareness efforts about the potential dangers of the unpredictable illicit drug supply and the intentional or unintentional co-use of substances, including antihistamines and opioids.

## Footnotes

*
https://www.elsevier.com/books/a-manual-of-adverse-drug-interactions/griffin/978-0-444-82406-6

† Adulterants are substances mixed with illicit drugs to lengthen the lifespan of the resulting product. Adulterants are pharmacologically active ingredients (e.g., caffeine and phenacetin).

§ SUDORS captures data on fatal unintentional and undetermined intent overdoses. For all captured overdose deaths, SUDORS records all drugs detected by postmortem toxicology, even those not ruled by a medical examiner or coroner as causing death. The medical examiner or coroner lists drugs on the death certificate based on any of the following: postmortem toxicology detection, evidence of drug use at the scene, or witness reports of drug use.

¶ Among 48 funded jurisdictions, 43 states and District of Columbia (DC) reported data during January 2019–December 2020. Twenty-six jurisdictions reported deaths during the entire period: Alaska, Connecticut, Delaware, DC, Georgia, Illinois, Kentucky, Maine, Massachusetts, Minnesota, Missouri, Nevada, New Hampshire, New Jersey, New Mexico, North Carolina, Ohio, Oklahoma, Pennsylvania, Rhode Island, Tennessee, Utah, Vermont, Virginia, Washington, and West Virginia. Eighteen additional jurisdictions reported deaths during at least one 6-month period during January 2019–December 2020 (i.e., January–June 2019, July–December 2019, January–June 2020, or July–December 2020): Alabama, Arizona, Arkansas, Colorado, Florida, Hawaii, Indiana, Iowa, Kansas, Louisiana, Maryland, Michigan, Mississippi, Montana, Nebraska, Oregon, South Dakota, and Wisconsin. Fifteen jurisdictions reported deaths from counties that accounted for ≥75% of drug overdose deaths in the state in 2017 for at least one 6-month period per SUDORS funding requirements (Alabama, Arkansas, Colorado, Florida, Hawaii, Illinois, Indiana, Louisiana, Michigan, Missouri, Nebraska, Pennsylvania, South Dakota, Washington, and Wisconsin); all other jurisdictions reported deaths from the full jurisdiction. Data were current as of April 25, 2022.

** Drug entries coded as antihistamines in SUDORS were allergy, allergy relief, antihistamines, Atarax, Benadryl, brompheniramine, carbinoxamine, cetirizine or cetirizine metabolite, chlorcyclizine or chlorcyclizine metabolite, chlorpheniramine or chlorpheniramine metabolite, cyproheptadine or cyproheptadine metabolite, desalkylhydroxyzine, descarboethoxyloratadine, desloratadine, dexbrompheniramine, dexchlorpheniramine, diphenhydramine or diphenhydramine metabolite, doxylamine or doxylamine metabolite, fexofenadine, hydroxyzine or hydroxyzine metabolite, hydroxyzine/cetirizine/meclizine (not distinguished) metabolite, levocetirizine, loratadine, mereprine, norchlorcyclizine, norcyproheptadine, nordiphenhydramine, norhydroxyzine norpromethazine, Nytol, phenergan, pheniramine, phenyltoloxamine, promethazine or promethazine metabolite, pyrilamine, tripelennamine, triprolidine, Unisom, Vistaril, and Zyrtec.

††
https://www2.census.gov/geo/pdfs/maps-data/maps/reference/us_regdiv.pdf

§§ Ninety-three percent of all SUDORS decedents in the 44 included jurisdictions had a toxicology report. Per SUDORS funding requirements, in a given 6-month period, ≥75% of SUDORS decedents in each individual jurisdiction had a toxicology report; 35 jurisdictions had toxicology reports for ≥90% of decedents in their jurisdiction in all included 6-month periods.

¶¶ IMFs include illicitly manufactured fentanyl and illicit fentanyl analogs, which were identified using both toxicology and scene evidence because toxicology alone cannot distinguish between pharmaceutical fentanyl and IMFs.

## References

[1] Mahdy AM, Webster NR. Histamine and antihistamines. Anaesth Intensive Care Med 2011;12:324–9. 10.1016/j.mpaic.2011.04.012

[2] Schifano F, Chiappini S, Miuli A, Focus on over-the-counter drugs’ misuse: a systematic review on antihistamines, cough medicines, and decongestants. Front Psychiatry 2021;12:657397. 10.3389/fpsyt.2021.657397 34025478 PMC8138162

[3] Banerji A, Long AA, Camargo CA Jr. Diphenhydramine versus nonsedating antihistamines for acute allergic reactions: a literature review. Allergy Asthma Proc 2007;28:418–26. 10.2500/aap.2007.28.3015 17883909

[4] Fiorentin TR, Krotulski AJ, Martin DM, Detection of cutting agents in drug-positive seized exhibits within the United States. J Forensic Sci 2019;64:888–96. 10.1111/1556-4029.13968 30485426

[5] O’Donnell J, Tanz LJ, Gladden RM, Davis NL, Bitting J. Trends in and characteristics of drug overdose deaths involving illicitly manufactured fentanyls—United States, 2019–2020. MMWR Morb Mortal Wkly Rep 2021;70:1740–6. 10.15585/mmwr.mm7050e3 34914673 PMC8675656

